# Mitochondria-associated membranes in aging and senescence: structure, function, and dynamics

**DOI:** 10.1038/s41419-017-0105-5

**Published:** 2018-02-28

**Authors:** Justyna Janikiewicz, Jędrzej Szymański, Dominika Malinska, Paulina Patalas-Krawczyk, Bernadeta Michalska, Jerzy Duszyński, Carlotta Giorgi, Massimo Bonora, Agnieszka Dobrzyn, Mariusz R. Wieckowski

**Affiliations:** 1 Department of Biochemistry, Nencki Institute of Experimental Biology, Warsaw, Poland; 2 Department of Morphology, Surgery and Experimental Medicine, Section of Pathology, Oncology and Experimental Biology, Laboratory for Technologies of Advanced Therapies (LTTA), University of Ferrara, Ferrara, Italy; 3 Departments of Cell Biology and Gottesman Institute for Stem Cell & Regenerative Medicine Research, Albert Einstein College of Medicine, Bronx, NY USA

## Abstract

Sites of close contact between mitochondria and the endoplasmic reticulum (ER) are known as mitochondria-associated membranes (MAM) or mitochondria-ER contacts (MERCs), and play an important role in both cell physiology and pathology. A growing body of evidence indicates that changes observed in the molecular composition of MAM and in the number of MERCs predisposes MAM to be considered a dynamic structure. Its involvement in processes such as lipid biosynthesis and trafficking, calcium homeostasis, reactive oxygen species production, and autophagy has been experimentally confirmed. Recently, MAM have also been studied in the context of different pathologies, including Alzheimer's disease, Parkinson’s disease, amyotrophic lateral sclerosis, type 2 diabetes mellitus and GM1-gangliosidosis. An underappreciated amount of data links MAM with aging or senescence processes. In the present review, we summarize the current knowledge of basic MAM biology, composition and action, and discuss the potential connections supporting the idea that MAM are significant players in longevity.

## Facts

- Contacts between mitochondria and the endoplasmic reticulum not only can be visualized by microscopic techniques but can also be isolated in order to investigate their protein and lipid composition.
- The molecular composition of the mitochondria-associated membranes (MAM) is closely related to its role in pivotal cellular processes.
- The involvement of the MAM fraction in numerous aging-associated pathologies has been confirmed.

## Open questions

- Are there any direct or indirect links between aging and MAM composition, function and dynamics?
- Which proteins present in the MAM could be involved in aging or senescence?
- Does the lipid composition of MAM change during aging-related processes?

## Introduction

**Fig. 1:**
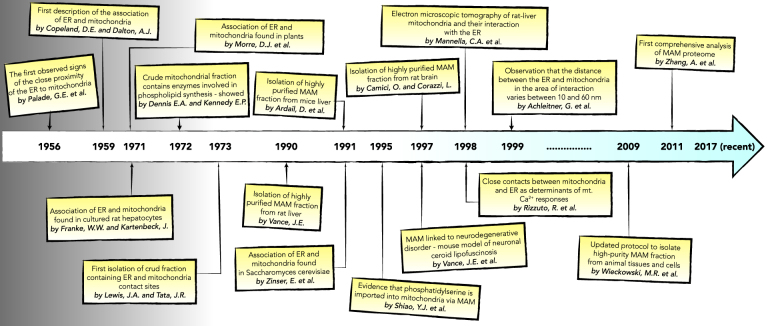
The historical timeline of the most important experimental observations and key discoveries in the course of studies devoted to interactions between mitochondria and the ER

Aging is a complex phenomenon related to gradual deterioration of cell, tissue, and whole organism functions throughout the lifespan. At the cellular level, aging was found to be associated with oxidative stress, accumulation of DNA modifications, impaired proteostasis, and inefficient organelle turnover^1,2^. Not surprisingly, aging affects function of individual organelles, including mitochondria and endoplasmic reticulum (ER), and thus, may also have impact on their contact sites. These contact sites can be identified as regions of biochemically distinct molecular composition, which are spatially restricted to the close vicinity of the interacting membrane fragments. The molecular assemblies forming such link provide a local environment, which can enhance the exchange of cargo or signals between organelles. Studies conducted in the past decades revealed that mitochondria-associated membranes (MAM) form a physical platform enabling communication between the ER and mitochondria, which is involved in lipid synthesis, Ca^2+^ trafficking and exchange (See Fig. 1)^3^. In addition, the proteome of the MAM fraction remains under intensive investigation in the context of different age-related disorders, such as Alzheimer's disease^4–8^, amyotrophic lateral sclerosis^9–11^, and type 2 diabetes mellitus^12,13^, as well as in obesity^14^, GM1-gangliosidosis^15^, and viral infection by human cytomegalovirus or hepatitis C virus^16,17^. Since the function of MAM has been better understood, different groups have tried to investigate their molecular composition and reveal which proteins found in MAM are truly transient or constantly present in MAM, as well as which molecules are simply a contamination caused by the imperfectness of used cell sub-fractionation protocols. In the present work, we describe close contacts between mitochondria and the ER following Giacomello’s and Pellegrini’s terminology, according to which isolated or purified membranes (involved in mitochondria-ER interactions) are referred to as the “***MAM fraction”***; however, when the architecture or ultrastructural organization of such contacts is discussed, we refer to them as mitochondria-ER contacts, “***MERCs***”^18^. In the present review we focus on the MAM proteome and its involvement in ROS production, lipid fluxes, autophagy, and regulation of Ca^2+^ turnover in senescence.

## MAM in aging and senescence: a proteomic perspective

The MAM proteome was comprehensively analyzed for the first time by Zhang et al.^19^, who identified 991 proteins in the “heavy” MAM fraction (which can be isolated at lower centrifugal forces compared to standard MAM isolation procedures). Later on, Poston et al.^20^ reported 1212 candidates, including weak soluble proteins, present at the MAM. Among them were commonly recognized MAM proteins: ACAT1, BiP/GRP78, calnexin, calreticulin, Erlin-1, Erlin-2, ERP44, HSPA9, MFN1, PDIA3, VDAC1, VDAC2, and VDAC3. The MS analysis enabled the characterization and classification of proteins identified in MAM into three groups: (1) those localized only in MAM (“MAM-resident proteins”); (2) those localized in MAM but present in other cellular compartments (“MAM-enriched proteins”); and (3) those temporarily present in MAM (“MAM-associated proteins”)^20^. Up to date, increasing number of reports has been published describing importance of the MAM proteome in regulation of cellular biology and senescence^17–23^.

### Mitochondrial structure and MERCs

Mitochondrial malfunctioning and structural variations have been linked with aging and age-associated disorders^21,22^. Mitochondrial morphology is very dynamic and can vary from a fragmented to a filamentous network as an effect of competition between the processes of fusion and fission, which are the key determinants of the mitochondrial quality control^23^. In particular, the levels of mitochondrial fusion proteins Mfn1 and Mfn2 were shown to be increased in aging skeletal muscle, indicating for upregulated fusion, likely in response to the accumulated mutations in the mitochondrial DNA^24,25^. The increased fusion was accompanied by reduced levels of the fission protein Fis1. Interestingly, mitochondrial network rearrangements are regulated by MERCs, which have been shown to mark the sites of mitochondrial fission^26^. Furthermore, senescent human adipose-derived mesenchymal stromal/stem cells exhibited increased levels of mitochondrial mass, superoxide and mitochondrial fusion proteins as mitofusin 1 (Mfn1)  and dynamin-related GTPase (OPA1) compared with young cells at low passages^27^. These observations indicate that changes in mitochondrial morphology observed in aging cells can be linked to the misregulated processes of fission and fusion.

### Misfolded protein aggregates present in MERCs

The loss of proteostasis, which is manifested by the decreased protein degradation ability of a cell, is one of the hallmarks of aging. Consequently, aggregates of damaged or misfolded proteins accumulate, leading to cell degeneration, and many pathologies. It has been recently reported that mitochondria are involved in the asymmetric segregation of the toxic aggregates during cell division in yeast^28–30^, which provides a mechanism for rejuvenation of the bud. In this process, the cellular debris is retained in the older mother cell, while the younger bud is essentially free of toxic protein waste. The protein aggregates have been shown to associate with the ER surface and localize at MERCs, indicating the possible role of MERCs in the protein quality control system^28^. A similar process was observed in immortalized human mammary epithelial stem-like cells undergoing asymmetric division, where newly synthesized mitochondria segregated preferentially to the daughter cell maintaining stemness properties, while daughter cells which received older mitochondria gave rise to differentiated cells^31^. Further studies using the split-GFP system in human RPE1 cells and in yeast revealed that cytosolic proteins prone to aggregation are imported into mitochondria in order to undergo degradation by mitochondrial proteases, such as Pim1^29^. This indicates that mitochondria play a role in both segregation and degradation of protein aggregates.

### Cooperation of mitochondria, the ER and MAM in ROS production

#### Reactive oxygen species (ROS) and aging

Increased intracellular levels of ROS and consequential oxidative damage to proteins, lipids, and DNA have been reported in many models of aging^32–34^. Although it is now clear that aging process is far too complex to be explained by one mechanism, the evidence that accumulation of oxidative damage is among the events contributing to aging phenomenon is quite extensive. Proteins responsible for intracellular ROS generation are located nearly in all subcellular compartments including mitochondria and the ER^34,35^. ROS present at moderate levels participate in intracellular signaling; however, excessive amount of these highly reactive molecules is harmful. Since MAM are dynamic structures enhancing communication between mitochondria and ER, they may play role in regulation of ROS production by ER and mitochondria.

#### ROS sources in mitochondria and ER

Mitochondrial respiratory chain has long been recognized as the main source of deleterious free radicals such as superoxide radical anion (O_2_^.•−^), which are responsible for age-related oxidative stress^36,37^. In recent years this view has been challenged and other intracellular ROS sources are gaining increased attention^38^. Depending on the tissue type, physiological state or pathological conditions, various enzymes localized in different subcellular compartments may be the dominant ROS producers. However, the significance of mitochondrial ROS in the aging process is supported by the marked overrepresentation of the mitochondrial proteome among the proteins subjected to oxidative damage throughout a lifespan^39^. The main ROS produced in mitochondria is superoxide radical anion O_2_^•−^, which is dismutated to H_2_O_2_. In turn, H_2_O_2_ gives rise to highly reactive OH in the reaction catalyzed by transition metals. There are several sites in mitochondria where ROS can be formed, including the respiratory chain complexes I and III. The rate of superoxide generation by these sites depends strictly on the redox state of the respiratory chain^33^. Other known mitochondrial ROS sources, releasing either O_2_^•−^ or H_2_O_2_, include the following: mitochondrial cytochrome b5 reductase^40^ and monoamine oxidases^41^ (associated with outer mitochondrial membrane), dihydroorotate dehydrogenase^42^, and glycerol-3-phosphate dehydrogenase (located at the outer surface of the inner mitochondrial membrane)^43^, electron transfer flavoprotein-ubiquinone oxidoreductase (localized on the matrix face of  the inner mitochondrial membrane), and two mitochondrial matrix enzyme complexes: α-ketoglutarate dehydrogenase^44,45^, and pyruvate dehydrogenase^35^. Interestingly, most of the abovementioned proteins and protein complexes have been found to be increasingly carbonylated during aging and senescence^39^.

When compared with mitochondria, ROS production in the ER is less studied, partly due to the limited choices of appropriate tools for measuring the ROS levels in this compartment. In the ER, proteins from the cytochrome P450 family^46^, NADPH oxidase 4 (Nox4)^47^, and endoplasmic reticulum oxireductin (Ero1)^48^ are the well-known ROS producers. Ero1 exists in two isoforms: Ero1-α and Ero1-β^49–51^. Interestingly, Ero1-Lα binds to the ER membrane especially in regions involved in MAM formation, and approximately 75% of Ero1-Lα is localized in the MAM fraction^52^. There is still missing evidence regarding ROS levels in the ER at different stages of life; however, aging appears to be accompanied by increased oxidative damage and the dysfunction of specific ER proteins, such as the ryanodine receptor (RyR)^53,^ the chaperones protein disulfide isomerase (PDI) and immunoglobulin heavy chain binding protein (BiP)^54,55^.

#### Mitochondria-ER contact sites as modulators of ROS synthesis and targets of oxidative damage

The MAM structure facilitates mitochondrial calcium uptake upon its release from the ER by coupling IP_3_R with a voltage-dependent anion channel (VDAC)^56^. The influx of Ca^2+^ to the mitochondrial matrix affects multiple aspects of mitochondrial function, such as Krebs cycle enzyme activity, ATP synthesis, mitochondrial permeability transition pore (PTP) opening, the mitochondrial membrane potential and respiration, and in consequence, mitochondrial ROS production^57–61^. Mutual dependencies between ER function and mitochondrial ROS production have also been demonstrated upon the aging-dependent deterioration of RyR function^53,59^. In the skeletal muscle of aged mice, increased carbonylation and cysteine nitrosylation of RyR1 was accompanied by channel “leakiness,” reduced Ca^2+^ transients upon electric stimulation of the muscle fibers, increased ROS levels and impaired muscle force production. The mitochondrially targeted overexpression of catalase diminished the oxidative modifications of RyR^59^. On the other hand, RyR1 destabilization by rapamycin treatment resulted in increased Ca^2+^ levels in the mitochondrial matrix, a decreased mitochondrial membrane potential and enhanced mitochondrial superoxide production^59^. Furthermore, increased mitochondrial lipid peroxidation in the skeletal muscle of mice with the Y522S mutation in RyR1 was associated with increased Ca^2+^ leakage through the channel^62^. Interestingly, mitochondrial damage, as well as accompanying muscle dysfunction, could be diminished by treatment with the antioxidant N-acetylcysteine, indicating involvement of ROS^62^.

The translocation and enrichment of the MAM fraction with the Ero1-Lα isoform is regulated by the oxidoreductive status of the ER environment. In fact, hypoxic conditions lead to the complete relocation of Ero1-Lα from MAM^52^. Ero1-Lα is a FAD-dependent oxidase that together with PDI plays an essential role in protein folding^63,64^. PDI directly interacts with newly synthesized and folded proteins and catalyzes disulfide bond formation by accepting electrons. In turn, Ero1 restores the oxidized state of PDI and transfers the accepted electrons from PDI to molecular oxygen, leading to H_2_O_2_ synthesis^64–66^. In addition, Ero1-Lα is crucial in the regulation of calcium release via MAM and IP3R1. During ER stress, Ero1-Lα oxidizes IP3R1, which potentiates the release of Ca^2+^ from the ER^49^. Next, ERp44 (ER luminal chaperone protein), which can also be found in MAM, binds to IP3R1, resulting in the inhibition of Ca^2+^ transfer to mitochondria at MERCs^67^. Interestingly, IP3R1 oxidation by Ero1-Lα causes the dissociation of ERp44 from IP3R1, thus promoting the activation of calcium release via IP3R1^49,68^.

**Fig. 2:**
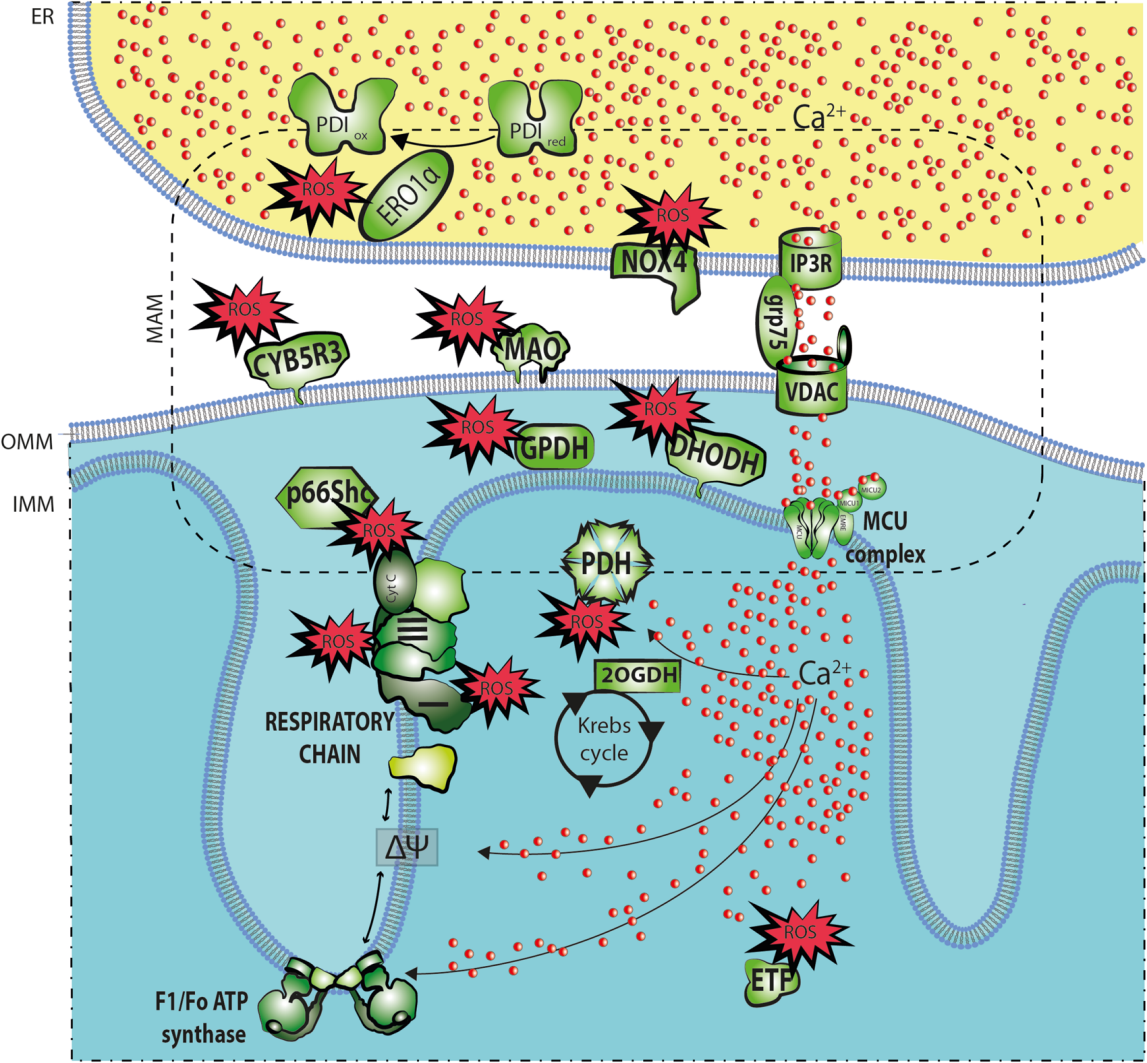
ROS-producing proteins localized in mitochondria, the ER, and MAM Schematic representation of ER, mitochondria, and MAMs with major mechanism of ROS production and Ca^2+^ cross-talk. 2OGDH Oxoglutarate dehydrogenase, CYB5R3 NADH:cytochrome b5 reductase, cyt. c cytochrome c, DHODH dihydroorotate dehydrogenase, Ero1 endoplasmic reticulum oxireductin, ETF electron transfer flavoprotein-ubiquinone oxidoreductase, Ero1α endoplasmic reticulum oxidoreductin, GPDH glycerol-3-phosphate dehydrogenase, GRP75 75 kDa glucose-regulated protein, NADH:ubiquinone oxidoreductase (I), CoQH2-cytochrome c reductase (III), IMM inner mitochondrial membrane, IP3R inositol triphosphate receptor, KGDHC α-ketoglutarate dehydrogenase complex, MAO monoamine oxidases A/B, Nox4 NADPH oxidase 4, OMM outer mitochondrial membrane, p66Shc p66Shc protein, PDI protein disulfide isomerase, PDH pyruvate dehydrogenase, VDAC voltage-dependent anion channel

Proteins present in MAM and involved in ROS generation are presented in Fig. 2.

#### P66Shc and its involvement in ROS production and aging

Among the many proteins found in the MAM, the 66-kilodalton isoform of the growth factor adapter Shc (p66Shc) protein has been reported to stimulate ROS synthesis and be tightly connected with the oxidative challenge, age-derived diseases and the aging process^69–71^. P66Shc together with p52Shc and p46Shc belongs to the ShcA family, and plays the role of a dominant negative regulator in the signal transduction from the growth factor receptor via the Ras-mediated signaling^72,73^. Furthermore, it has been demonstrated that p66Shc knockout mice are less sensitive to oxidative and hypoxic stress and live approximately 30% longer than wild-type animals^69^.

While p66Shc is considered a cytosolic protein, it has also been found in the following locations: (a) the mitochondrial matrix^74^; (b) the mitochondrial intermembrane space^70^; (c) associated with the OMM from its cytosolic side^71^; and finally (d) in the MAM fraction. Exogenous or endogenous oxidative stress can stimulate the critical phosphorylation of p66Shc at the Ser36 residue^69^ and enhance its translocation to or association with mitochondria^75^. The p66Shc is phosphorylated at Ser36, and subsequently isomerized, dephosphorylated, and finally translocated to the mitochondrial intermembrane space (MIMS) and/or the MAM fraction, where it participates in ROS production^70,75–80^. The p66Shc catalyzes the reduction of O_2_ to H_2_O_2_ in the mitochondrial intermembrane space at the cost of cytochrome c oxidation, which appears to be an important step in the induction of apoptosis through the mitochondrial pathway^70^. Unfortunately, whether p66Shc is translocated to the MIMS in mitochondria^70^ or binds to the OMM (from the cytosolic side) involved in MAM formation^71^ remains a matter of debate. Yet, regardless in which cellular compartment p66Shc contributes to ROS production^81^, its participation in the feedback loop of ROS-induced p66Shc ROS production indicates that p66Shc could be involved in mammalian lifespan regulation. Thus, by translating oxidative stress damage into cell death, p66Shc becomes an apoptotic inducer shortening the lifespan^75^. The *p66Shc* mRNA and p66Shc protein were highly expressed in fibroblasts from centenarians compared with fibroblasts from young and elderly individuals^82^. In contrast, the primary cultures of skin fibroblasts derived from newborn and 18-month-old mice expressed similar levels of p66Shc^71^. However, the expression of p66Shc was significantly higher in the liver, heart, lungs, skin, and diaphragm of adult mice than in newborn littermates^69^. Higher levels of p66Shc in the MAM isolated from the livers of old mice and increased ROS production by crude mitochondria (containing MAM) argue in favor of the translocation of p66Shc to the MAM in the cellular response to age-related oxidative stress^71,83^. Moreover, p66Shc is also present in plasma membrane-associated membranes (PAM). Interestingly, the level of p66Shc changes reciprocally in PAM and MAM, depending on the age of the animal^71^.

**Fig. 3:**
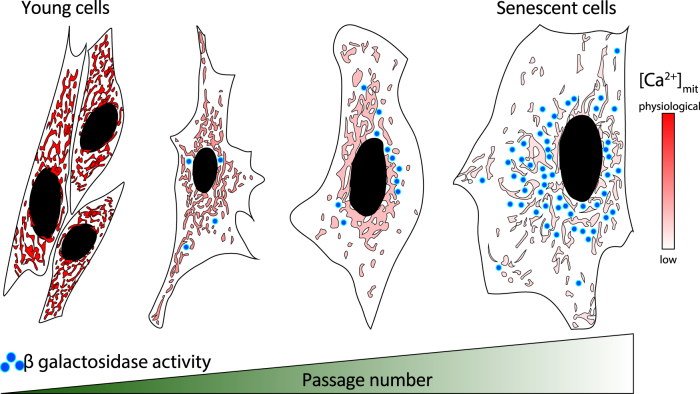
Mitochondrial calcium uptake as a function of passage number Mitochondrial Ca^2+^ responses ([Ca^2+^]_mit_) in MEFs during ATP challenge as a function of a passage number. The pseudocolor scale (on the *right*) indicates the approximate changes of mitochondrial calcium level, where light pink represents low Ca^2+^ and red high (physiological) Ca^2+^ levels. Blue dots—represent senescence marker, β-galactosidase activity

It has been demonstrated that an extracellular agonist-stimulated Ca^2+^ uptake by mitochondria in mouse embryonic fibroblasts (MEFs) is gradually decreased with culture time (see Fig. 3)^75^. Interestingly, such dependency was not reported in p66Shc-deficient MEFs^75^. After oxidative challenge, a reduction in the mitochondrial Ca^2+^ response and fragmentation of the three-dimensional mitochondrial network was observed in wild-type MEFs, but only minor changes in the Ca^2+^ response and morphology were detected in p66^Shc–/–^ cells^75^. Moreover, the inhibition of p66Shc phosphorylation at Ser36 with the use of hispidin, a specific blocker of the PKCβ isoform, preserved the mitochondrial morphology in wild-type MEFs. Similarly, no alterations in the passage-dependent decrease in mitochondrial calcium were observed in these cells after treatment with hispidin^75^.

#### MAM, the link between mitochondria and the ER in mitochondrial Ca^2+^ uptake in senescent cells

Studies of a neuronal aging model revealed increased Ca^2+^ transfer from the ER to mitochondria in long-term cultured neurons, whereas no functional coupling was observed between the ER and mitochondria during short-term culturing^84^. The increased Ca^2+^ uptake by mitochondria is considered to be responsible for the downregulation of store-operated calcium entry, which in turn causes the impaired stability of mushroom spines, leading to aging-associated cognitive decline^84^. The increased ER-mitochondria Ca^2+^ transfer was accompanied by the upregulation of the mitochondrial calcium uniporter (MCU)^85^, which suggests the involvement of MERCs in the process, since they are hotspots for Ca^2+^ signaling^86,87^. Increased Ca^2+^ transfer to mitochondria could serve as a regulatory mechanism to counterbalance the loss of mitochondrial potential in aging cells. The proposed mechanism of the Ca^2+^ flux through MERCs involves control over the calcium channel expression level as well as the number and structure of MERCs. Indeed, the number of contact sites is a well-known determinant of the extent of Ca^2+^ transferred between mitochondria and ER^88,89^. The mechanism of such regulation relies on the laws of diffusion, according to which doubling the distance causes a fourfold increase in the travel time required, thus reducing the efficiency of diffusional transport at larger distances^18^. Recently, it was demonstrated that ultrastructure of the MERCs itself, in particular the thickness of MERCs, is a crucial factor regulating the efficiency of Ca^2+^ transport^18^. Interestingly, knockdown of MCU and inositol 1,4,5-trisphosphate receptor type 2 (ITPR2), both involved in the accumulation of calcium in mitochondria, resulted in senescence escape, indicating the role of mitochondrial calcium accumulation in senescence induction^90^. Similarly, lower number of contacts between mitochondria and the ER in senescent human fibroblasts could be also responsible for the compromised mitochondrial calcium uptake in senescent cells. Notwithstanding this, additional studies are needed to identify which factors have the highest influence of the regulation of Ca^2+^ fluxes through MERCs in aging cells.

## MAM and longevity: a lipidomic perspective

Morphological data indicate that MERCs are a critical platform for direct interorganelle lipid synthesis and rapid lipid transit^91^. In fact, MAM formation, integrity and functioning depend on tightly regulated lipid species and a flexible, yet unique, proteome^92^.

### Structural composition and dynamic role of MAM finally come of age

**Fig. 4:**
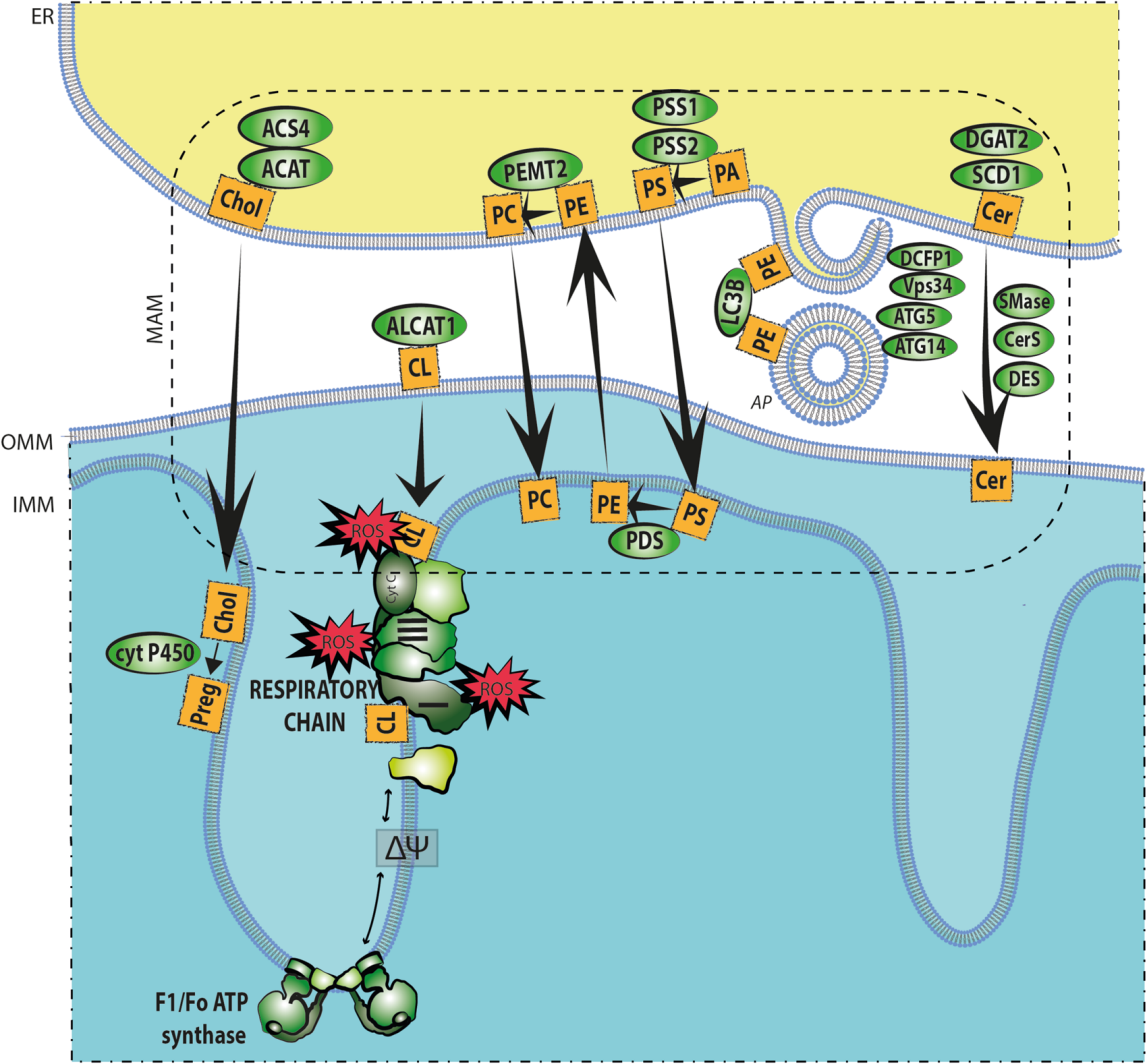
Lipid network at the MAMs The ER-mitochondria contact sites integrate assembly of autophagosomes, synthesis, and trafficking of phospholipids, cholesterol (Chol) and ceramides (Cer) by a network of MAM- residing enzymes. AP autophagosome, LC3B microtubule-associated protein 1 light chain 3, CL cardiolipin, PA phosphatidic acid, PS phosphatidylserine, PE phosphatidylethanolamine, PC phosphatidylcholine, Preg pregnolone

In comparison to the bulk of the ER, MAM are characterized by an increased thickness due to their reinforcement with cholesterol and sphingolipids. Additionally, MAM are characterized by a different degree of curvature, phospholipid composition, and degree of unsaturation^7^. As a consequence, the disruption of MAM integrity and MAM malfunction are linked to an aberrant metabolism and a decreased lifespan. Hence, not surprisingly, MAM are enriched with several lipid transfer proteins and biosynthesis enzymes, including acyl-CoA:cholesterol acyltransferase/sterol *O*-acyltransferase 1 (ACAT1/SOAT1), diacylglycerol *O*-acyltransferase 2 (DGAT2), phosphatidylserine synthases 1 and 2 (PSS1 and PSS2), phosphatidylethanolamine N-methyltransferase 2 (PEMT2), fatty-acid CoA ligase 4 (FACL4/ACS4), fatty-acid transport protein 4 (FATP4), and stearoyl-CoA desaturase 1 (SCD1) (See Fig. 4)^93–98^.

Initially, MAM were recognized as domains enriched in enzymes of the phospholipid biosynthesis and remodeling pathway^99^. Indeed, phosphatidylserine (PS) is synthesized in the ER by the MAM enzymes PSS1 and PSS2. The newly formed PS is transferred to the outer surface of the mitochondrial inner membrane via MAM, where it is converted into phosphatidylethanolamine (PE) by phosphatidylserine decarboxylase. Subsequently, PE returns to the ER, where PEMT2 mediates the synthesis of phosphatidylcholine (PC). The serine exchange activity is catalyzed by both enzymes, PSS1 and PSS2, whereas PSS1 governs the exchange of choline exclusively^91,100^. Nevertheless, the transfer of PS into mitochondria through MAM is the rate-limiting step during the generation of PE^91^.

In addition, MAM accommodate enzymes indispensable for cholesterol biosynthesis^101–104^. The intracellular conversion of free cholesterol to cholesteryl esters is catalyzed by ACAT1 in order to coordinate the dynamic equilibrium between membrane-bound and cytoplasm-stored cholesterol in a resting state^105^. However, during a stress response, cholesterol import to mitochondria is sustained where cytochrome P450 initiates steroidogenesis^101^. Moreover, the depletion of cholesterol in MAM was found to favor the association between MAM and mitochondria and lead to not only a decline in the de novo synthesis of PS but also an improvement in PE synthesis^101^.

Since the proteome of MAM contains sphingomyelin phosphodiesterase (SMase), ceramide synthase (CerS), and dihydroceramide desaturase (DES), a certain pool of ceramides is believed to be produced at the aforementioned contact sites^102,103,106^. Importantly, due to the proapoptotic character of ceramides in mitochondria, MAM might represent a critical checkpoint for preventing ceramide influx, hence regulating shifts in the cellular lifespan.

### The commitment of MAM and autophagy to lifespan regulation

In order to promote longevity, protection against cell damage and death is also mediated through autophagy, with special regards of the macroautophagy class. Macroautophagy (hereafter referred as autophagy) is recognized as a catabolic process that degrades and recycles the bulk of cytosolic components and organelles in response to cellular stress and bioenergetic demands^107,108^. The formation of a double-layered structure known as an autophagosome (AP), is a mandatory hallmark of autophagy. The AP sequesters components and then fuses with lysosomes in order to deliver its cargo for degradation by lysosomal proteases and hydrolases^109^. Basal autophagy levels are indispensable for physiological quality control, but the impairment and declined efficacy of autophagy have been implicated in numerous human pathologies and aging^110^.

Since the discovery of autophagy, there has been intensive debate regarding the membrane and lipid donor source, which is necessary for the expansion and maturation of the AP. The membranes of mitochondria, the ER, golgi apparatus, and PM, and fairly recently, MAM, have been proposed to contribute to AP assembly^107,111^. The abundance of autophagy-related proteins (ATG), including ATG5, declines in the brains of aging individuals^112^. Under starvation, the omegasome marker DCFP1 (double FYVE domain-containing protein 1), the pre-AP marker ATG14, Vps34 and ATG5, proteins that are critical for AP formation, relocalize toward the MAM fraction^111^. Disruption of the interaction between ATG14 and DCFP1 in MAM by the knockdown of *Pacs2* and *Mfn2* in cells prevented proper AP formation and downstream microtubule-associated protein 1 light chain 3 (LC3) lipidation^113^. In agreement with this model, disruption of MERCs by the ablation of *Mfn2* in human cancer cell lines inhibits interorganelle lipid transfer and starvation-induced autophagy by halting the PS trafficking between the ER and mitochondria-derived APs^113^. Moreover, the abundance of mitochondria-derived PE and PS corresponds to longevity^107,114^.

More recently, a role of lipid rafts in regulating autophagy induction was defined in primary human and mouse embryo fibroblasts^115^. The gangliosides account for paradigmatic lipid raft constituents^116^. The GD3 ganglioside was reported to participate in AP biogenesis and maturation by molecular association with key modulators of autophagic vacuoles, including LC3-II, PtdIns3P, LAMP1, AMBRA1, and BECN1^115,117^. Moreover, GD3 was reported to be enriched in ER-mitochondria-associated membranes^118^, also upon autophagic stimulation^115^. In addition, lipid rafts were confirmed at the MAM location during autophagic sequelae^115^. Hence, aforementioned data favor the hypothesis that MAMs operate as a functional platform for early steps of the AP formation, thus any disturbances in the MAMs action and integrity are potentially transitioned into impaired autophagy.

The membrane theory of aging supports the idea that lifespan is inversely related to unsaturated membrane PL content^119^. Caloric restriction (CR) without malnutrition is the most effective strategy for inducing autophagy and the key anti-aging intervention for extending the lifespan of yeast, flies, and mice^110^. Concordantly, CR results in a decrease in the percentage of n-3 and an increase in the percentage of n-6 polyunsaturated fatty acids (PUFA)^120^, but the ratio of n-3:n-6 PUFAs decreases with increasing lifespan. Such a decrease in membrane PUFA and a reduced degree of unsaturation contribute positively to the aging process by lowering susceptibility to peroxidative damage^22^. Moreover, MAM-enriched SCD1 is a critical lipid metabolism enzyme that regulates the cellular ratios of saturated/monounsaturated fatty acids (MUFA), and thus remains fundamental for the structure of cellular membranes^121^. The gene expression of *DGAT2*, which co-localizes with SCD1 in the ER^98^, was reported to decrease in the skin of aging individuals^122^. In line with this notion, the inhibition of SCD1 impaired AP biogenesis and affected AP fusion with lysosomes^123,124^. Diminished SCD1 activity was associated with alterations in the status of cellular membrane PE, PC, PS, and cardiolipin (CL) accumulation, composition, and saturation^124^. Furthermore, MAM-delivered PL are critical contributors to ATG protein activation and of autophagy sequela initiation^109^.

The depletion of CL and its pathological remodeling coincide with aging. In turn, these changes affect MAM structure and function^107^, and CL transfer was proposed to depend on MAM^125^. Unlike other PLs, CL is found almost exclusively in the mitochondrial inner membrane, where it governs the organization and assembly of respiratory complexes^126^, as well as is involved in control of the mitochondrial fission machinery^127,128^. In eukaryotic tissues, CL contains MUFA or di-unsaturated chains with 16–18 carbons^107^, predisposing CL to be more oxidative stress-susceptible. In fact, the CL fatty acids were remodeled from linoleic acid (18:2n-6) to more unsaturated acids, such as arachidonic (20:4n-6) and docosahexaenoic (22:6n-3), in aged rats^129^. One of the enzymes involved in CL remodeling is MAM-enriched acyl-CoA:lysoCL acyltransferase 1 (ALCAT1), which in pathological conditions, remodels CL with acyl-CoAs enriched in long-chain highly unsaturated fatty acids^107^. Consequently, ALCAT1^−/−^ mice were protected from the onset of age-related diseases, including obesity, type 2 diabetes and hepatosteatosis^107^.

### Time flies: defects in MAM couples to aberrant autophagy during neurodegeneration

Dysregulation of autophagic flux leads to accumulation of abnormal protein aggregates and deteriorated organelles, which alongside reduced expression of ATG, are commonly observed in aging^130^. Hence, age is the greatest risk factor for the development of neurodegenerative disorders such as Parkinson’s disease (PD) or Alzheimer’s disease (AD)^131^.

The PD-related proteins, Parkin, and PTEN-induced novel kinase 1 (PINK1) are involved in mitochondrial recycling and sequester damaged mitochondria for autophagic clearance by mitophagy^132^. Moreover, mutations in Parkin and PTEN were associated with familiar and sporadic cases of PD^130,133^ and MAMs were identified as the prime location for local recruitment of LC3-II and a membrane source for the mitophagosome^134^. The α-synuclein (SNCA) is another factor contributing to degeneration of dopaminergic neurons in familiar and sporadic PD incidents^135^. The aberrant aggregation of SNCA into oligomers during PD is limited by the chaperone-mediated autophagy^136^. The majority of SNCA resides in cytoplasm; however, a subpopulation of SNCA was found in MAM^137^, and its overexpression increased the extent of contact sites and MAM activity^137,138^. Furthermore, PD-associated mutant forms of human SNCA exhibited diminished binding to MAM and disrupted ER-mitochondria tethers^137^.

In AD pathology, sequential proteolytic cleavage of amyloid precursor protein (APP) releases toxic amyloid β peptides (Aβ)^93^. MAM were shown to be the major site of the Aβ formation, since APPs and the majority of the γ-secretase localized to the MAM^4,139,140^, followed by enlarged ER-mitochondria contact area and increased MAM functionality^5^. In fact, upregulation of several MAM-associated lipid metabolism enzymes, including ACAT1^5^, was reported in human AD brain cortical tissue, APP_Swe/Lon_ mice, and primary neurons exposed to Aβ^141^. Genetic or pharmacological blockage of ACAT1 increased APs formation and diminished amyloidopathy in brains of young and old transgenic AD mice^142^. Moreover, significant elevation of membrane- and autophagic vacuole-derived lipid species, including cardiolipin, gangliosides, or cholesteryl esters was observed alongside exacerbated Aβ levels in cellular systems, AD mouse models and AD individuals^143,144^. Hence, a plethora of evidence points to tightly regulated composition and dynamics of MAM lipids as a requirement during autophagy and cellular lifespan, but the underlying molecular mechanisms of such relationship remain a matter of intense investigation.

## Concluding remarks

In the current biological perspective, a direct link between the molecular composition of MAM and aging remains highly underappreciated and awaits further scientific attention. The following indirect evidence supports the assumption that MAM significantly impact cellular function and longevity: (a) the cell passage-dependent gradual decrease in mitochondrial calcium uptake and the lower number of MERCs in senescent cells; (b) the association between the abundance of p66Shc protein in MAM and animal lifespan; (c) the importance of MAMs in regulation of lipid fluxes and autophagy, and (d) the enrichment of MAMs with the proteins that are involved in the development of age-related neurological and metabolic disorders. Whether some of the described proteins are truly localized in MAM or their presence in MAM fraction results from imperfectness of the fractioning techniques remain matter of intense debate. Nevertheless, targeting MAM structure, function, and dynamics might expand the therapeutic repertoire for numerous disease conditions, as well as sustained longevity.
